# Ovarian follicular responses to estrus and ovulation synchronization protocols in East African Shorthorn Zebu cattle

**DOI:** 10.1186/s13028-025-00821-w

**Published:** 2025-07-28

**Authors:** Patrick Mawadri, Tonny Balemwa, Denis Rwabiita Mugizi, Patrick Vudriko, Benon Mbabazi Kanyima, Charles Lagu, David Okello-Owiny, James Okwee-Acai, Maria Gorretti Nassuna-Musoke

**Affiliations:** 1 Department of Veterinary Pharmacy, Clinical and Comparative Medicine, School of Veterinary Medicine and Animal Resources, College of Veterinary Medicine, Animal Resources and Biosecurity, Makerere University, P. O Box 7062, Kampala, Uganda; 2 Lanoa Agricultural and Technology Consult Limited, P.O. Box 1805, Mbarara, Uganda; 3 Department of Animal Production and Range Management, Faculty of Agriculture and Environment, Gulu University, P. O. Box, 166, Gulu, Uganda

**Keywords:** *Bos indicus*, Estrus, Ovulation, Reproductive technology, Breeding, Tropical cattle fertility, Fixed-time insemination, Infertility, Follicular dynamics

## Abstract

**Background:**

The East African Shorthorn Zebu (SHZ) is a *Bos indicus* breed adapted to tropical farming systems characterized by compromised feeding, welfare and harsh environments, contributing to poor reproductive performance. Estrus and ovulation synchronization protocols developed to enhance reproduction in *Bos taurus* have not achieved similar success in SHZ. This study evaluated effects of 7-day Co-synch + progesterone releasing intravaginal device (7-day Co-synch + P4ID; n = 17), Bee-Synch I (n = 17), and Bee-Synch II (n = 17) protocols on dominant follicle (DF) diameters, estrus and ovulation in 51 SHZ cows on extensive management. Estrus was monitored through observation and aids, while ultrasonography measured ovarian DF and corpus luteum (CL) diameters. Generalized linear models were used to compare means across protocols at 95% confidence level using R.

**Results:**

DF diameters increased by 0.05 mm for every hour from P4ID withdrawal irrespective of protocol (P < 0.001). Mean DF diameters at P4ID withdrawal were 6.015 ± 0.903, 4.93 ± 0.737 and 7.31 ± 0.613 mm for 7-day Co-synch + P4ID, Bee synch I and Bee synch II respectively and the difference between Bee synch I and Bee synch II were significant (P.adj = 0.044). At last gonadotropin-releasing hormone (GnRH) administration time, DF diameters were 8.76 ± 0.725, 7.29 ± 0.505, and 9.68 ± 0.521 mm for 7-day Co-synch + P4ID, Bee synch I and Bee synch II respectively, with significant differences between Bee-Synch I and Bee-Synch II (P-adj = 0.016). Mean preovulatory DF diameters were 10.64 ± 0.333, 8.97 ± 0.335 and 10.30 ± 0.236 mm for the 7-day Co-synch + P4ID, Bee synch I and for Bee synch II respectively, with significant differences between Bee-synch I and 7-day Co-synch + P4ID (P.adj = 0.011) and between Bee-Synch I and Bee-Synch II (P.adj = 0.008). Estrus expression rates were not significantly different and 47%, 41.2% and 58.8% for 7-day Co-synch + P4ID, Bee synch I and Bee synch II respectively, while ovulation rates were 41.2%, 52.94% and 82.35% respectively. Mean ovulation times and last GnRH to ovulation intervals were significantly longer for both 7-day Co-synch + P4ID and Bee synch II than for Bee synch I.

**Conclusions:**

Bee synch II and 7-day Co-synch + P4ID outperformed Bee synch I presenting opportunities for fertility improvement in SHZ. The results highlight the need for tailored fixed-time estrus and ovulation synchronization protocols to enhance fertility in *B. indicus* cattle under tropical conditions.

## Background

The East African Short-horn Zebu (SHZ) cattle, a major breed in Uganda, are known for their high heat tolerance and disease resistance. However, they have low milk yields, slow growth rates, and small carcass weights, typical of tropical *Bos indicus* breeds [1–3]. Assisted reproductive technologies, such as artificial insemination (AI), in vitro fertilization, embryo transfer, and cryopreservation, offer affordable and efficient selection-based breeding options to improve SHZ genetics and productivity [4].

AI is vital for genetic progress in Africa, as high-grade male cattle are often inaccessible or unaffordable for most farmers [3]. Hormonal estrus synchronization has further optimized AI by overcoming challenges such as labor-intensive heat detection and enabling simultaneous breeding of multiple cows [5, 6]. However, most estrus synchronization protocols were initially designed for *Bos taurus* cattle. Significant differences between *B. taurus* and *B. indicus* cattle with regards to female reproductive physiology, which is the basis for the development of estrus and ovulation synchronization protocols have been documented. For example, in *B. indicus* cows, the duration of estrus and the interval between onset of estrus and ovulation have wide variations [7]. Additionally, *Bos indicus* cows exhibit silent or split estrus, low estrus signs, and high rates of anestrus, which affect the effectiveness of protocols for estrus and ovulation synchronization [8]. While these characteristics depict the existing unique adaptive diversity of *Bos indicus* cattle and provide opportunities for sustainable improvement of livestock productivity, the differences justify development of protocols for estrus synchronization and ovulation tailored to mitigating the requirement for estrus detection and to inducing anestrus cows to fertility among *Bos indicus* within a specific geographical location [9].

Various protocols that combine gonadotrophin releasing hormone (GnRH), prostaglandin (PG) and progesterone releasing intravaginal devices (P4ID), in short (GnRH-PG-P4ID) protocols have been developed to prevent estrus and ovulation from the day of P4ID insertion to the day of its removal and to induce estrus in anestrous cows [10]. Such protocols provide a breakthrough for managing infertility arising from poor estrus detection, improper timing of insemination, anovulation and delayed ovulation, traits that characterize the SHZ in the less developed tropical farming systems [7, 8]. Disappointingly, while these GnRH-PG-P4ID protocols have been successful in *B. taurus* cows [6, 11], they have yielded lower conception rates in *B. indicus* and crossbred *B. indicus* x *B. taurus* cows [12, 13].

The 7-day Co-synch + P4ID involves insertion of an intravaginal P4ID on day 0 concurrent with an injection of GnRH. On day 7 the P4ID is withdrawn, concurrent with an injection of PG. Cows are then inseminated 60–66 h post P4ID withdrawal, with an additional dose of GnRH. The rationale is that in the presence of high concentrations of progesterone from the P4ID, the GnRH dose on day 0 induces regression rather than ovulation of a follicle if present, leading to formation of a corpus luteum (CL). This first GnRH dose concurrently stimulates formation of a new follicular wave at the beginning of the progesterone treatment on day 0, and since the normal duration of a follicular wave is 7–10 days, withdrawal of the P4ID on day 7 will ensure there is no persistent follicle formed. During the entire duration of the P4ID, ovulation and estrus expression are prevented, allowing time for growth and maturation of the CL to a state fully responsive to the PG action given on day 7 [10]. In order to improve conception rates and efficiency the 7-day Cosynch + P4ID was modified to emerge with two protocols i.e., Bee Synch I and Bee Synch II. Studies on ovarian follicular patterns and conception in crossbred *B. indicus* x *B. taurus* individuals in the United States have reported higher pregnancy rates with these protocols in *B. indicus* cattle breeds [14–16]. The Bee synch I protocol is designed to mitigate the formation of persistent follicle by reducing duration of P4ID administration and consequently, the age of the follicle from 7 to 5 days [14]. It incorporates a PG dose at the onset of the treatment to cause CL regression in those animals with a mature CL, lower the plasma progesterone levels and increase frequency of LH pulses leading to increased speed of follicular maturation during the treatment period. A double dose of PG on day 5 is administered to guarantee regression of an immature day 5 CL. Insemination is conducted 62–66 h post progesterone withdrawal. The Bee synch II is a modification of Bee synch I protocol derived by eliminating the GnRH injection at the protocol onset and administration of only a single dose of PG on day 5. Elimination of the first GnRH dose is aimed at removing the possibility that an immature CL would be present on day 5 of the protocol, and thereby concurrently eliminating the requirement for double dose of PG and generally reducing the costs involved in the GnRH [14–17].

Our recently unpublished survey identified the 7-day Co-synch + P4ID as the most commonly used progesterone-based fixed-time AI protocol in Uganda. There is however no literature on its performance in Uganda. Given genotypic variation between *B. indicus* cattle breeds plus the difficult environmental and less than optimal management conditions, further research is needed to evaluate the response of the Ugandan SHZ to the three Co-synch + P4ID protocols. This study aimed to evaluate the effects of Bee-Synch I and Bee-Synch II protocols on follicular dynamics and estrus in the SHZ cows, in comparison to the 7-day Co-synch + P4ID protocol. We tested the hypothesis that reducing P4ID implant duration from 7 to 5 days with (Bee synch I) or without (Bee synch II) an additional dose of PG during implant insertion and withdrawal, will not lead to differences in DF diameters, the occurrence and timing of estrus and ovulation, compared to the original 7-day Co-synch + P4ID protocol.

## Methods

### Study location

The study was conducted from August to December 2022 on Lusenke Stock Farm belonging to the National Animal Genetic Resources Center and Data Bank, a government body mandated with animal breeding in Uganda. It is located in Kayunga District in Central Uganda (Latitude 0.882827, longitude 32.983797, altitude 1048 m). Ambient temperature was 16–30 °C with an average precipitation of 100.7–232.8 mm during the study period.

### Experimental subjects

The subjects were non-pregnant postpartum suckled SHZ cows, maintained on an extensive management system. Cows were overseen by herdsmen to graze in paddocks comprised mainly of star grass (*Cynodon nlemfuensis*), *Chloris gayana* and *Pennisetum purpurium* with plenty of tree shades and watering points. Vitaphos Maziwa mineral block (Cibus Animal Nutrition Ltd, Nairobi, Kenya) was available to the cows in the paddocks. The farm conducted anthelmintic deworming and trypanosomiasis prophylaxis every 3 months. Acaricide application was done every week using a spray race to control ticks and tick-borne diseases. All experimental subjects were screened and were free from brucellosis, campylobacteriosis, and trichomoniasis.

Breeding was exclusively by artificial insemination. Heat signs were observed by herdsmen. Only animals that had been recorded to have exhibited heat signs of standing to be mounted or vaginal mucus discharge or activated Estrotect® patches at least once in the prior 2 months were selected and forwarded for gynecological ultrasound examination. Animals that were confirmed non-pregnant with normal reproductive organs and cycles by presence of either a corpus luteum or growing follicles ≥ 4 mm diameter and subsequent were included in the experiment. Furthermore, the body condition score (BCS) and weights of the experiment subjects were measured the day before initiation of the experiment treatments. BCS ranged from 2.5 to 4, on a scale of 1–5, where 1 meant very thin and 5 very fat [18]. The body weights of experiment cows were normally distributed with a mean of 264.8 ± 4.22 kg, and ranged from 198 to 345 kg.

### Experimental design

East African Shorthorn Zebu cows (n = 51) were divided into three treatment groups, 7-day Co-synch + P4ID (n = 17), Bee Synch I (n = 17) and Bee Synch II (n = 17). The sample size was arrived at using power analysis at 95% level of confidence, 80% statistical power, and using Cohen’s *d* effect size of 0.5 [19] adjusted for 10% attrition [20]. BCS was used to randomly allocate the experimental subjects to the three groups such that each group consisted of 2 cows with BCS 2.5, 8 cows with BCS 3.0, 4 cows with a score of 3.5, and 3 cows with a score of 4.0. Cows in each group (n = 17) comprised parities ranging from 1 to 5, grouped into primiparous (parity 1) and multiparous (parity 2–5). Group 1 comprised 3 primiparous and 14 multiparous cows while groups 2 and 3 were each composed of 6 primiparous and 11 multiparous cows. The mean weights of cows in the 3 experiment groups were similar and were 257.29 ± 6.61, 274.82 ± 8.49, and 260.18 ± 6.35 kg for groups 1, 2, and 3 respectively {F (2,48) = 1.699, P = 0.194}. Group 1 was assigned 7-day Co-synch + P4ID, while Group 2 was allocated to Bee Synch I and Group 3 to Bee Synch II.

### Estrus and ovulation synchronization protocols

**Fig. 1:**
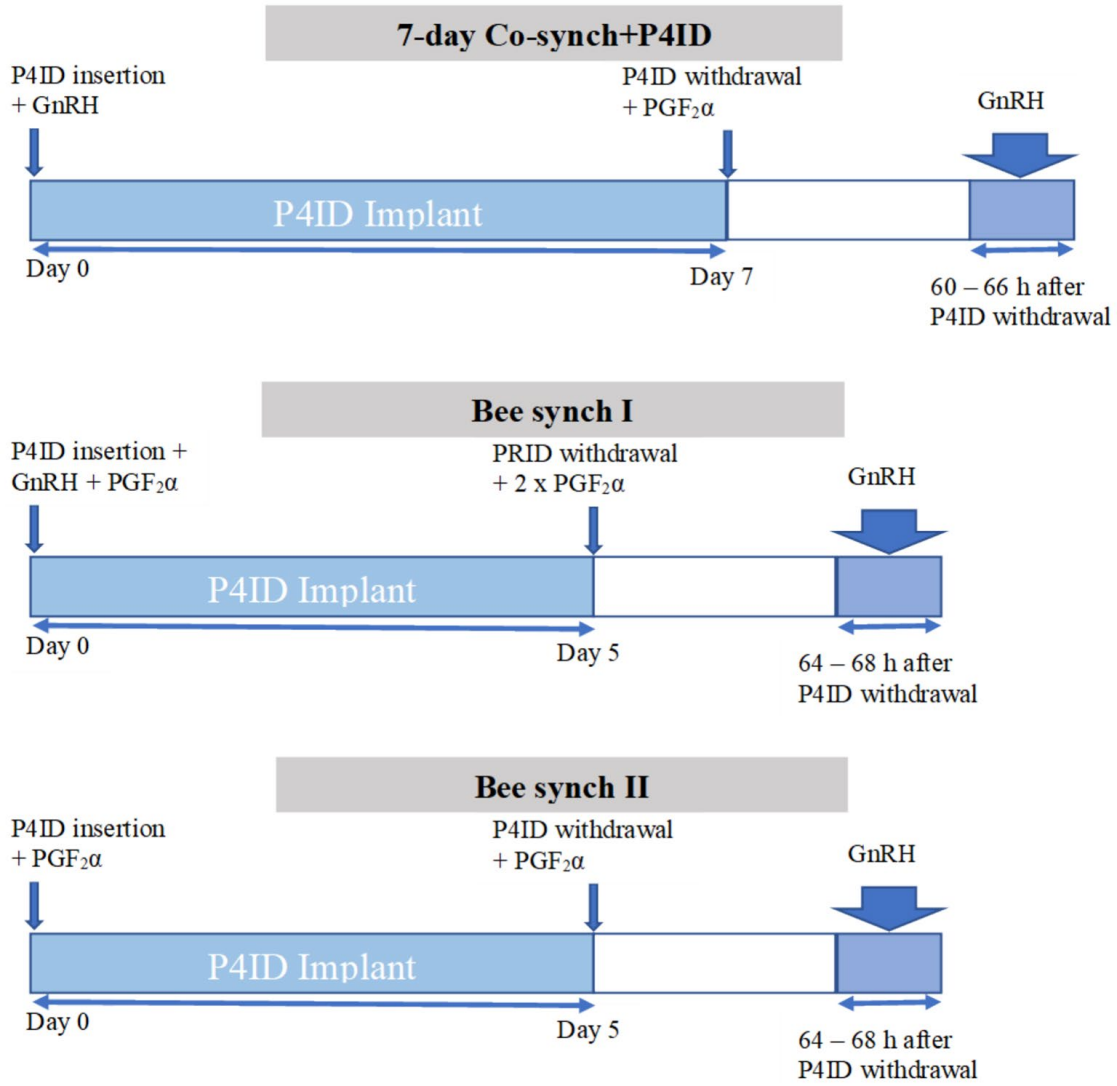
Estrus and ovulation synchronization treatment protocols. At the onset of estrus and ovulation synchronization (day 0), all cows in each group received a P4ID (PRID^®^ delta, 1.55 g). In addition, cows in the 7-day Co-synch + P4ID group received a dose of GnRH (gonadorelin, 100 mcg), those in the Bee synch I group received a dose of GnRH and PGF_2_α (dinoprost, 25 mg) while the Bee synch II treatment group received a dose of PGF_2_α. The P4ID implant was withdrawn on day 7 coupled with a single dose of PGF_2_α for 7-day Co-synch + P4ID. On the other hand, the P4ID implant was withdrawn on Day 5 together with a double dose of PGF_2_α for Bee synch I and a single dose of PGF_2_α for Bee synch II. No AI was conducted for all groups

Cows in the 7-day Co-synch + P4ID group received a dose (100 mcg) of Gonadorelin (Cystorelin, Ceva, Libourne, France) concurrently with the P4ID implant (PRID^®^ delta, Kansas -USA, containing 1.55 g of progesterone) on day 0. This was followed by the administration of a dose (25 mg) of prostaglandin F_2_α (PGF_2_α)—dinoprost (Enzaprost^®^, Ceva, Libourne, France) on day 7 concurrent with P4ID withdrawal. An additional dose (100 mcg) of GnRH was given 63 h (h) from the time of P4ID withdrawal [14, 17]. In the Bee synch I group, cows received a dose each of GnRH (gonadorelin 100 mcg) and PGF_2_α (dinoprost 25 mg) concurrently with P4ID insertion on day 0. This was followed by the administration of a double dose of PGF_2_α (dinoprost 50 mg) on Day 5 together with P4ID withdrawal. A second dose of GnRH (gonadorelin 100 mcg) was administered 66 h from the time of P4ID withdrawal [16]. In the Bee synch II group, a dose of PGF_2_α (dinoprost, 25 mg) was administered concurrently with P4ID insertion on day 0. This was followed by a single dose of PGF_2_α (dinoprost, 25 mg) administered on Day 5 together with P4ID withdrawal. A dose of GnRH (gonadorelin, 100 mcg) was administered 66 h from the time of P4ID withdrawal [16]. The days mentioned are all in reference to the day of the start of each synchronization exercise, considered as day 0 (Fig. 1). The treatment of subjects in each of the three experiment groups was conducted serially rather than together to provide adequate time for accurate data collection by trans-rectal ultrasonography. Each subject was fitted with an estrus detection patch, A.I. TAGS™ (Cotran Europe, Comines, France) attached between the hip and the tail head and placed perpendicularly over the spine on the day of P4ID withdrawal.

### Determination of ovarian parameters by ultrasound examinations

Every cow in each experiment group was subjected to trans-rectal ultrasonographic examination to detect the presence or absence of a dominant follicle (DF) and corpus luteum (CL). The ultrasonographic examinations were conducted by one researcher throughout the experiment. Ovarian transrectal ultrasound evaluations were performed with an ultrasound Scanner; Anyscan Multi®, 2019 (SONGKANG GLC Co ltd, Seongnam City, Gyeonggi-do Province, South Korea) equipped with a 6.5 MHz rectal linear probe. The diameter of each DF and CL was taken and calculated as the average of one measurement on the longitudinal axis and another on the transverse axis and these were taken at right angles using electronic calipers in positions where the corpus luteum or dominant follicle showed maximum diameters on frozen images [21, 22].

The ultrasonographic examinations were performed by the same technician during the study, starting at the initiation of each protocol and then on the respective days of P4ID withdrawal. Thereafter, the examinations were conducted twice daily, at an interval of 12 h for four consecutive days and the occurrence of ovulation was marked by the absence of a DF ≥ 7 mm diameter between two consecutive examinations [23]. Ovulation was confirmed by the presence of a CL during another ultrasonographic examination conducted 5 days after last dose of GnRH for each subject where the presence or absence of a corpus luteum on the ipsilateral side of detected ovulation was checked and measurements taken for this CL (Day 5 CL diameter) if present. The ‘ovulation time’ was the time interval from P4ID withdrawal to ovulation occurrence, calculated by subtracting 6 h from the time interval between P4ID withdrawal and ultrasonographic ovulation detection [23, 24].

The ‘estrus onset to ovulation interval’ was recorded as the time interval (h) from estrus detection to ovulation occurrence. The DF diameters were captured during the twice daily ultrasonographic examination that were conducted at 12 h interval, starting on the days of P4ID withdrawal and proceeded for the next 4 days. The DF diameters at the time P4ID withdrawal were referred to as the ‘DF diameters at P4ID withdrawal’. The DF diameters were also recorded around the time of administration of GnRH 63 h after progesterone withdrawal from the 7-day Co-synch + P4ID group and 66 h for both Bee synch I and Bee synch II groups, referred to as the ‘Last GnRH to ovulation interval’. The ‘preovulatory DF diameters’ were the diameters of the dominant follicles as recorded during the ultrasound examination preceding the one that detected its disappearance.

### Determination of estrus expression

Each cow was observed twice daily for estrus signs for 5 days, beginning on the day of P4ID withdrawal and for 1 h each in the morning (06:00–07:00 h) and evening (18:00–19:00 h). A cow was recorded to be on heat when it stood to be mounted and/or its estrus detection patch became activated by an undetected standing mount. The estrus detector was considered activated when there was appearance of the orange dye on at least 50% of the patch, covering the central midline area [15, 25]. The interval between P4ID withdrawal and detection of the first standing heat was recorded as the ‘estrus onset time’.

### Statistical analyses

The raw data were entered into a Microsoft Excel spreadsheet and later exported to R version 4.4.2 [26]. Descriptive statistics including graphs were generated using R studio. For each outcome variable, the data for each protocol group were visualized using histograms and density plots and measures of central tendency and dispersion obtained using the ‘describe’ function from the ‘psych’ package in R [27] and each group was tested for normality using Shapiro Wilk tests at 95% confidence level to guide in model selection of analysis tools.

A linear mixed model was used to predict the effects of protocol and time from P4ID withdrawal on DF diameters. The “lme” function from the “nlme” package in R [28] was used to fit a linear mixed model with fixed effects for protocol, Hours and their interaction, and with a random intercept for subject (Model 1: lme (DF diameter ~ Protocol * Hours, random =  ~ 1 | subject, data = data). The final model was selected after several F tests to compare the random and fixed effects of the full and reduced models and lowest AIC and Bayesian information criterion (BIC) values. The selected model was checked for fitness using the ‘qqnorm’ function from the “ggstatsplot” package [29] and the ‘check_model’ function from the “performance” package in R [30].

DF diameters at P4ID withdrawal time, DF diameters at time of last GnRH dose and preovulatory DF diameters were normally distributed continuous data (P > 0.05) in each of the protocol groups, and Linear models using the ‘glm’ function from the ‘stats’ package in R [26] was used, with family = “Gaussian” and link = “identity”, to compare their mean values across protocols at 95% confidence level. The models used were: Model 2 (glm(DF diameter at P4ID withdrawal ~ Protocol + Parity, family = Gaussian (link = identity)), Model 3 (glm(DF diameter at last GnRH ~ Protocol + BCS + Parity, family = Gaussian (link = identity)), and Model 4 (glm (Preovulatory DF diameter ~ Protocol + BCS + Parity, family = Gaussian (link = identity)). Estrus onset time, ovulation time, estrus onset to ovulation interval and last GnRH to ovulation intervals were positive count data (discrete number of hours) that were non-normally distributed (P < 0.05) in at least one of protocol groups and their data demonstrated overdispersion (variance > mean). Negative binomial models were therefore used for analyses using the ‘glm.nb’ function from the “MASS” package in R [31]. These models include: Model 8 (glm.nb (Estrus onset time–Protocol + DF diameter at_P4ID withdrawal), Model 9 (glm. nb (Ovulation time–Protocol), Model 10 (glm. nb (Last GnRH to ovulation interval–Protocol + Parity), and Model 11 (glm. nb (estrus onset to ovulation interval–Protocol). Day-5 CL diameters were continuous data, non-normally distributed but with left-skewness and under dispersion (variance < mean) and the Gamma model with “log” link was used {Model 5 = glm (Day-5 CL diameter–Protocol, family = Gamma (link ="log"). R^2^ = coefficient of determination; AIC = Akaike Information Criterion; * represents statistically significant p-values; ^Φ^estimates in original scale}.

During analysis, parity and BCS were initially included in each model and the Akaike Information Criterion (AIC) values were compared between models and selected models were checked for model fitness using the ‘simulateResiduals’ function in the “DHARMa” package in R [32]. In each case, the model which had the lowest AIC and a satisfactory goodness-of-fit was retained. Post hoc pairwise comparisons were conducted using the ‘pairs’ and ‘emmeans’ functions from the ‘emmeans’ package in R [33] were used to compute estimated marginal means and facilitate pairwise comparisons, and the adjust = “non” and adjust = “holm” were used to obtain raw and adjusted p-values respectively while the argument, type = “response” was used to back-transform estimates to the original scale where generalized linear models like negative binomial or Gamma models were used.

## Results

### Variation of dominant follicle (DF) diameters with time (hours) from P4ID withdrawal

**Fig. 2:**
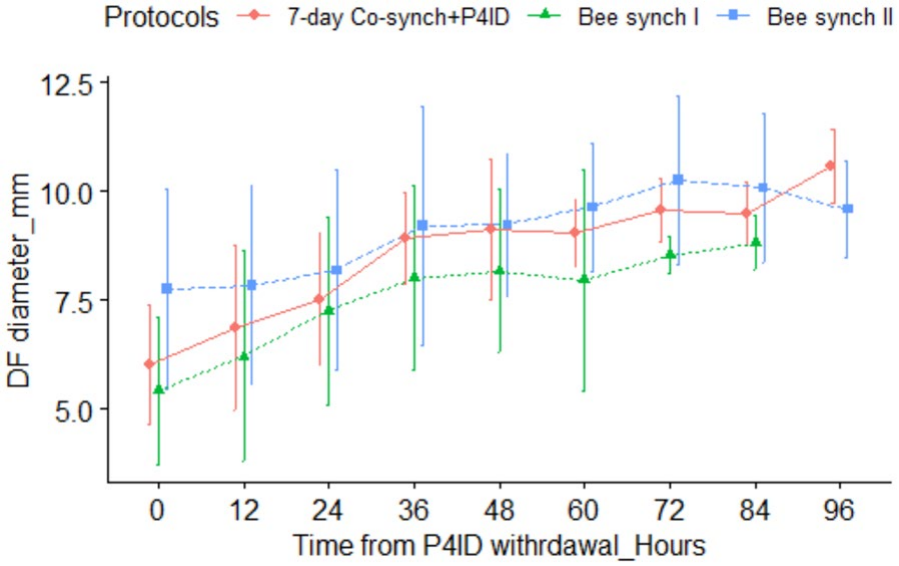
Follicular diameter variation with time from P4ID withdrawal. Variation of DF diameter with time following P4ID withdrawal, showing general increase in DF diameter with time across the three estrus and ovulation synchronization protocols

There was an observed increase in DF diameter with time, from the time of P4ID withdrawal up to ovulation time, across the three protocols tested (Fig. 2). There was a significant effect of time (Hours) from P4ID withdrawal on DF diameter (P < 0.001), indicating a directly proportional relationship. For every 1 h increase from the time of P4ID withdrawal, the DF diameter increases by 0.05 mm on average, irrespective of the treatment protocol. Compared to the Bee Synch I protocol, the 7-day Co-synch + P4ID protocol did not show a statistically significant difference in DF diameter at baseline (Estimate = 0.633 mm, P = 0.390). Bee Synch II showed a trend toward greater baseline DF diameter (Estimate = 1.208 mm), though this difference did not reach statistical significance (P = 0.069). Interaction terms between Protocols and Hours were not statistically significant, indicating that the rate of follicular growth over time did not differ meaningfully across protocols. These findings suggest that while follicular diameter increases significantly with time, synchronization protocol does not substantially alter the rate of this increase (Table 1).

**Table 1:** Effects of Protocol and time from P4ID withdrawal on DF diameters

Variable	Estimate	SE	z-value	P-value
Bee synch I–7-day Cosynch + P4ID	− 0.632	0.724	− 0.873	0.390
Bee synch II–7-day Cosynch + P4ID	0.575	0.656	0.877	0.388
Bee synch II–Bee synch I	1.208	0.638	1.892	0.069
Hours	0.047	0.005	8.406	< 0.001*
Bee synch I**:** Hours	0.0008	0.0085	0.099	0.921
Bee synch II**:** Hours	− 0.0084	0.0067	− 1.249	0.213
7-day Cosynch + P4ID**:** Hours	− 0.0008	0.0085	− 0.099	0.921

There was a significant main effect of time (Hours), with DF diameter increasing by 0.047 mm per hour (P < 0.001), while the main effects of protocols and the Protocol x Hours interaction effects were non-significant (P > 0.05)

Estimate, estimated marginal means; SE, standard error

Model 1: lme (DF diameter ~ Protocol x Hours, random =  ~ 1**|**subject); Akaike Information Criterion (AIC) = 740.95; Coefficient of determination (R^2^) = 0.74

*represents statistically significant P-values

### Dominant follicle (DF) diameter at the time of P4ID withdrawal

The mean DF diameter at time of P4ID withdrawal and other ovarian response parameters across the three protocols are presented in Table 2. The effect of parity was significant, indicating that DF diameters for the pluriparous cows were by 1.9 mm larger than for primiparous cows (P = 0.032). Similarly, DF diameters for the cows in the Bee synch II group were significantly higher than for cows in the Bee synch I group (P.adj = 0.044). However, there were no statistically significant differences in DF diameters between Bee synch I and 7-day Cosynch + P4ID groups (P.adj = 0.761) and between Bee synch II and 7-day Co-synch + P4ID groups (P.adj = 0.435; Table 3).

**Table 2:** Variation of DF diameters, estrus and ovulation parameters across protocols in SHZ cows

Variables	7-day Co-synch + P4ID	Bee-Synch I	Bee-Synch II
	*Mean ± SE*		
DF diameter at P4ID withdrawal (mm), n = 30	6.01 ± 0.9.3	4.93 ± 0.737	7.31 ± 0.613
DF diameter at last GnRH dose (mm), n = 30	8.76 ± 0.725	7.29 ± 0.505	9.68 ± 0.521
Pre-ovulatory diameter (mm), n = 30	10.64 ± 0.333	8.97 ± 0.335	10.30 ± 0.236
Day 5 CL diameter, n = 30	13.2 ± 0.823	10.67 ± 0.591	14.0 ± 0.663
Mean estrus onset time (h)^Ꝑ^	69.0 ± 6.09	61.7 ± 6.51	61.2 ± 5.44
Mean ovulation time (h)^Ꝑ^	96.9 ± 3.27	78.0 ± 2.63	97.7 ± 2.33
Mean estrus onset to ovulation interval (h)	32.0 ± 6.58	15.0 ± 8.06	39.0 ± 5.70
Last GnRH to ovulation interval (h)	33.9 ± 3.00	12.0 ± 3.00	29.7 ± 2.12
	*n (% within protocol)*		
Estrus expression, n = 17 per group	8 (47.00)	7 (41.18)	9 (52.94)
Ovulation, n = 17 per group	8 (47.00)	9 (52.94)	14 (82.35)
	*n (% across protocols)*		
Ovulation with estrus, n = 20	7 (35)	5 (25)	8 (40)
Ovulation with no estrus, n = 11	1 (9.1)	4 (36.4)	6 (54.5)
Estrus with no ovulation, n = 6	1 (16.7)	3 (50)	2 (33.3)
No estrus and no ovulation, n = 14	8 (57.14)	5 (35.72)	1 (7.14)

Variation of DF diameters at P4ID withdrawal, at last GnRH dose and preovulatory DF diameters, as well as estrus and ovulation parameters across the studied protocols in SHZ cows

DF diameters were generally smaller for the Bee synch I protocol compared to 7-day Cosynch + P4ID and Bee synch II protocols

While estrus expression rates were similar, ovulation was more frequent in the Bee synch II protocol than others

A high percentage of cows had ovulation after estrus occurrence compared with those that did not show estrus

^Ꝑ^represents the time (hours) from progesterone (P4ID) withdrawal

**Table 3:** Effects of protocol, BCS and parity on DF diameters and Day-5 CL diameter

Contrast	Estimate	SE	P-value	P.adj
DF diameter at P4ID withdrawal time *(Model 2; R*^*2*^ = *0.34, AIC* = *125.3)*				
Bee synch I–7-day Cosynch + P4ID	− 0.763	1.089	0.490	0.761
Bee synch II–7-day Cosynch + P4ID	1.709	1.027	0.109	0.435
Bee synch II–Bee synch I	2.472	0.889	0.010	0.044*
Pluriparous–Primiparous	1.912	0.842	0.032*	
DF diameter at time of last GnRH dose *(Model 3; R*^*2*^ = *0.52, AIC* = *105.37)*				
Bee synch I–7-day Cosynch + P4ID	− 1.134	0.827	0.185	0.371
Bee synch II–7-day Cosynch + P4ID	0.979	0.757	0.211	0.371
Bee synch II–Bee synch I	2.113	0.677	0.005	0.016*
BCS 3–BCS 2.5	1.273	1.010	0.224	0.447
BCS 3.5–BCS 2.5	2.699	1.050	0.0187	0.103
BCS 4–BCS 2.5	3.502	1.350	0.017	0.103
BCS 3.5–BCS 3	1.427	0.651	0.040	0.162
BCS 4–BCS 3	2.230	1.190	0.075	0.224
BCS 4–BCS 3.5	0.803	1.210	0.514	0.514
Pluriparous–Primiparous	1.56	0.649	0.026*	
Preovulatory DF diameters *(Model 4; R*^*2*^ = *0.70, AIC* = *73.239)*				
Bee synch I–7-day Cosynch + P4ID	− 1.360	0.442	0.006	0.011*
Bee synch II–7-day Cosynch + P4ID	− 0.114	0.376	0.765	0.765
Bee synch II–Bee synch I	1.246	0.367	0.003	0.008*
BCS 3–BCS 2.5	1.595	0.527	0.006	0.019*
BCS 3.5–BCS 2.5	2.736	0.571	< 0.001	< 0.001*
BCS 4–BCS 2.5	3.091	0.711	< 0.001	0.001*
BCS 3.5–BCS 3	1.141	0.345	0.003	0.014*
BCS 4–BCS 3	1.495	0.622	0.025	0.050*
BCS 4–BCS 3.5	0.355	0.655	0.594	0.594
Pluriparous–Primiparous	1.08	0.35	0.006*	
Day 5 CL diameter *(Model 5; AIC* = *142.77)*^Φ^				
Bee synch I–7-day Cosynch + P4ID	− 2.601	1.010	0.015	0.031*
Bee synch II–7-day Cosynch + P4ID	0.869	1.060	0.418	0.418
Bee synch II–Bee synch I	3.470	0.888	< 0.001	0.002*

Effects of protocol, BCS and parity on DF diameters at the times of P4ID withdrawal, last GnRH dose and preovulation, and Day-5 CL diameters of SHZ cows. Whereas DF diameter at P4ID withdrawal and at last GnRH dose were affected by only protocol, preovulatory DF diameters were affected by protocol, BCS and parity. Preovulatory DF diameters were significantly larger for pluriparous than primiparous cows (P = 0.006)

Estimate = estimated marginal means, SE = standard error of estimate; Adjustment method = Holm. Model 2 = glm(DF diameter at P4ID withdrawal ~ Protocol + Parity, family = Gaussian (link = identity)); Model 3 = glm(DF diameter at last GnRH ~ Protocol + BCS + Parity, family = Gaussian (link = identity)); Model 4 = glm (Preovulatory DF diameter ~ Protocol + BCS + Parity, family = Gaussian (link = identity)); and Model 5 = glm (Day-5 CL diameter ~ Protocol, family = Gamma (link ="log")

R^2^, coefficient of determination; AIC, Akaike Information Criterion

*Represents statistically significant p-values

^Φ^Estimates in original scale

### DF diameter at the time of the last GnRH injection

**Fig. 3:**
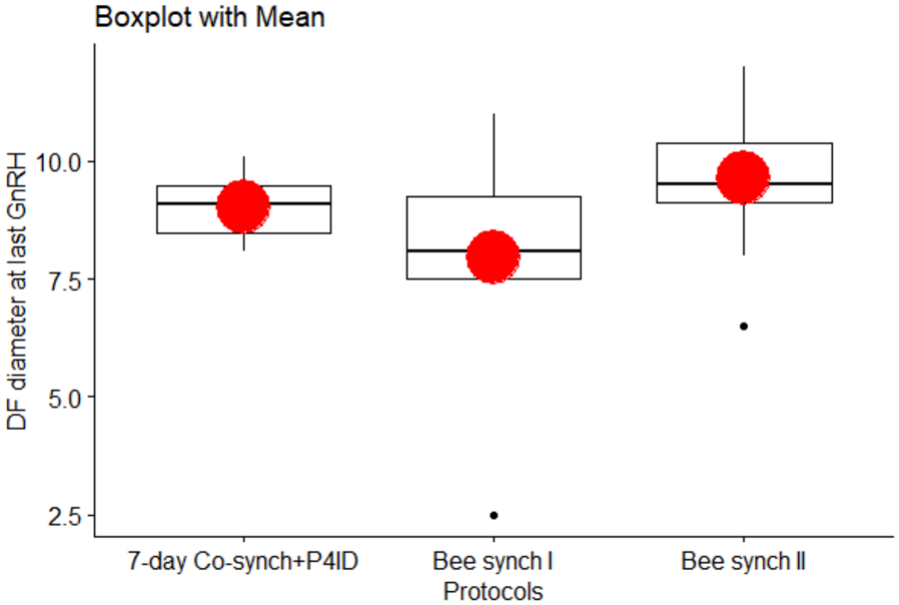
DF diameter at last GnRH for the three protocols. The mean DF diameters were higher for both 7-day Co-synch + P4ID and Bee synch II protocols compared to that for Bee synch I protocol

The DF diameters at the time of GnRH administration also varied across the synchronization protocols and as presented in Table 2 and Fig. 3. The effect of parity was significant, indicating that the DF diameters at last GnRH dose were by 1.6 mm higher in pluriparous cows than in primiparous cows (95% CI [0.29, 2.83], t(20) = 2.40, P = 0.016). The results revealed that DF diameters at last GnRH dose were by 2.1 mm higher in cows in the Bee synch II group than cows in the Bee synch I group (P.adj = 0.016) but there were no significant differences between 7-day Cosynch + P4ID and either Bee synch I or Bee synch II groups (P > 0.05). Cows with BCS 3.5 and 4.0 tended to having higher DF diameters compared to each of the lower BCS of 2.5 and 3.0 but these differences were not significant after “holm” adjustments (P > 0.05; Table 3).

### Preovulatory DF diameter

**Fig. 4:**
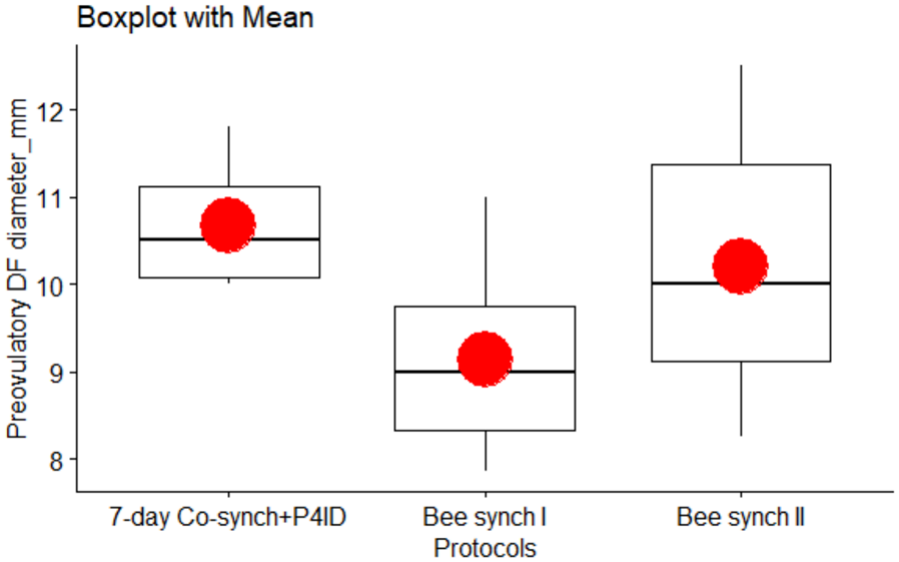
Preovulatory DF diameters for cows in the three protocols. The mean preovulatory diameter was significantly higher for both 7-day Co-synch + P4ID and Bee synch II protocols compared to that for Bee synch I protocol

The mean (± standard errors) pre-ovulatory DF diameters are summarized in (Table 2 and Fig. 4). The effects of protocol were significant, indicating that the preovulatory DF diameters were by 1.36 mm larger in the 7-day Co-synch + P4ID group than the Bee synch I group (P.adj = 0.011). Preovulatory DF diameters were also by 1.25 mm on average, larger in the Bee synch II group than in the Bee synch I group (P.adj = 0.008) while there were no significant differences between 7-day co-synch + P4ID and Bee synch II groups (P.adj = 0.765). Similarly, cows in BCS 3.0, 3.5 and 4.0 at protocol onset had significantly larger preovulatory DF diameters than cows in BCS 2.5, by 1.6, 2.7 and 3.1 mm (P.adj = 0.019, < 0.001 and 0.001) respectively. The DF diameters were also by 1.1 mm and 1.5 mm significantly larger in cows with BCS 3.5 (P.adj = 0.013) and 4.0 (P.adj = 0.05) respectively, compared to cows in BCS 3.0, while there were no significant differences between cows in BCS 3.5 and BCS 4.0 (Table 3).

### Day 5 CL diameter

The mean diameters of the Day 5 CL are presented in Table 2. Day-5 CL diameters were significantly larger for the 7-day Co-synch + P4ID group than for Bee synch I group by 2.6 mm (SE = 1.01, P.adj = 0.031), and by 3.5 mm larger for the Bee synch II group than the Bee synch I group (P.adj = 0.001). However, there was no significant difference between Bee synch II and 7-day Co-synch + P4ID groups (P.adj = 0.418; Table 3).

### Estrus expression and ovulation rate

Generally, the ovulation rate was higher than the estrus expression rate irrespective of the synchronization protocols used (Table 2). The odds of estrus expression were 21.3% lower for cows in the Bee synch I group compared to the 7-day Cosynch + P4ID group, while the odds were by 60% higher for cows in the Bee synch II group compared to 7-day Cosynch + P4ID group. Similarly, the odds of estrus expression are twice as high among cows in the Bee synch II group as cows in the Bee synch I group. However, all these differences were not statistically significant (P > 0.05; Table 4). Bee synch II protocol was associated with significantly higher odds of ovulation detection compared to 7-day Cosynch + P4ID (OR = 22.94; P.adj = 0.029). The difference between Bee synch II and Bee synch I protocols was marginally non-significant (OR = 8.45; P.adj = 0.079). No significant difference was observed between Bee synch I and 7-day Cosynch + P4ID protocols (P.adj = 0.3257). These findings suggest a potential relative effectiveness of Bee synch II protocol in enhancing ovulation. The odds of ovulation tended to be higher in pluriparous cows than primiparous cows but this difference was marginally non-significant (OR = 7.7, SE = 0.152, z = 1.024, P = 0.081). Pairwise comparisons of estimated marginal means revealed no statistically significant differences in ovulation detection across BCS levels after adjusting for multiple comparisons (Holm correction). While the contrast between BCS 3 and BCS 4 showed a trend (OR = 20.0, P = 0.1251), the overall lack of significant findings may be due to limited sample sizes or separation in some BCS groups, as indicated by zero or inflated odds ratios with infinite or undefined standard errors.

**Table 4:** Effects of protocols and cow parity on estrus expression and ovulation

Contrast	OR^Φ^	SE	P-value	P.adj
Estrus expression (*Model 6; AIC* = *75.578)*				
Bee synch I/7-day Cosynch + P4ID	0.787	0.545	0.936	
Bee synch II/7-day Cosynch + P4ID	1.6	0.431	0.772	
Bee synch II/Bee synch I	2.04	0.341	0.562	
Ovulation occurrence (*Model 7: AIC* = *53.48)*				
Bee synch I/7-day Cosynch + P4ID	2.71	2.76	0.326	0.326
Bee synch II/7-day Cosynch + P4ID	22.73	0.053	0.009	0.029*
Bee synch II/Bee synch I	8.47	0.12	0.039	0.079
BCS2.5/BCS3	0.00	0.00	0.308	1.0000
BCS2.5/BCS3.5	0.00	0.00	0.994	1.0000
BCS2.5/BCS4	6.00	8.00	0.205	1.0000
BCS3/BCS3.5	0.00	0.00	0.994	1.0000
BCS3/BCS4	20.00	26.00	0.021	0.1251
BCS3.5/BCS4	1.02 × 10^1^⁰	2.71 × 10^13^	0.993	1.0000
Pluriparous/Primiparous	7.69	0.152	0.081	

Whereas there was no significant variation in the odds of estrus expression between protocols, ovulation had 22.7 times higher odds of occurrence in Bee synch II group than 7-day Cosynch + P4ID group (P.adj = 0.029)

The effects of BCS and parity on ovulation were not statistically significant

OR, odds ratio in original scale; SE, standard error; Adjustment method, Holm

Model 6 = glm (Estrus expression ~ Protocol, family = binomial (link = “logit”))

Model 7 = glm (Ovulation occurrence ~ Protocol + Parity + BCS, family = binomial (link = “logit”))

R^2^, coefficient of determination; AIC, Akaike Information Criterion

*Represents statistically significant p-values

^Φ^Estimates are in original scale

### Estrus onset time

The mean estrus onset times for the synchronization protocols are presented in Table 2. There were no significant differences in estrus onset time among the Protocol groups (P > 0.05). While Bee synch I appeared to have a slightly shorter onset time than 7-day Cosynch + P4ID (10.6% or 7.2 h shorter), and 7-day Cosynch + P4ID slightly longer estrus onset time than Bee synch II (12.7% or 7.8 h longer), none of these differences reached statistical significance (Table 5).

**Table 5:** Effects of protocols and parity on estrus and ovulation times

Contrast	Ratio^Φ^	SE	P-value	P.adj
Estrus onset time (Model 8; R^2^ = 0.49, AIC = 155.37)				
Bee synch I/7-day Cosynch + P4ID	0.894	0.120	0.406	
Bee synch II/7-day Cosynch + P4ID	0.887	0.139	0.329	
Bee synch II/Bee synch I	0.992	0.130	0.948	
Ovulation time (Model 9; R^2^ = 0.72, AIC = 203.68)				
Bee synch I/7-day Cosynch + P4ID	0.806	0.071	< 0.001	< 0.001*
Bee synch II/7-day Cosynch + P4ID	1.01	0.047	0.851	0.851
Bee synch II/Bee synch I	1.25	0.063	< 0.001	< 0.001*
Last GnRH to ovulation interval (Model 10; R^2^ = 0.74, AIC = 220.97)				
7-day Cosynch + P4ID/Bee synch I	3.09	0.645	< 0.001	< 0.001*
Bee synch II/7-day Cosynch + P4ID	0.850	0.139	0.333	0.333
Bee synch II/Bee synch I	2.64	0.492	< 0.001	< 0.001*
Pluriparous – Primiparous	1.34	0.193	0.044*	
Estrus onset to ovulation interval (Model 11; R^2^ = 0.48, AIC = 153.19)				
7-day Cosynch + P4ID/Bee synch I	2.13	0.707	0.022	0.045*
Bee synch II/7-day Cosynch + P4ID	1.22	0.323	0.455	0.455
Bee synch II/Bee synch I	2.60	0.818	0.002	0.007*

Pairwise contrasts of estrus and ovulation times. While there were no significant differences in estrus onset time, ovulation time was shorter in Bee synch I compared to either 7-day Cosynch + P4ID or Bee synch II protocols

Last GnRH—ovulation and estrus onset—ovulation intervals were also significantly longer in both 7-day Cosynch + P4ID and Bee synch II protocols than for Bee synch I protocol, and last GnRH—ovulation interval was longer in pluriparous than primiparous cows

Ratio, Mean time ratio in original scale; SE, standard error; Adjustment method, Holm

Model 8 = glm.nb (Estrus onset time–Protocol + DF diameter at_P4ID withdrawal; Model 9 = glm. nb (Ovulation time–Protocol)

Model 10 = glm. nb (Last GnRH to ovulation interval–Protocol + Parity)

Model 11 = glm. nb (estrus onset to ovulation interval–Protocol); R^2^, coefficient of determination; AIC, Akaike Information Criterion

^Φ^Estimates are in original scale

*Represents statistically significant P-values

### Ovulation time

**Fig. 5:**
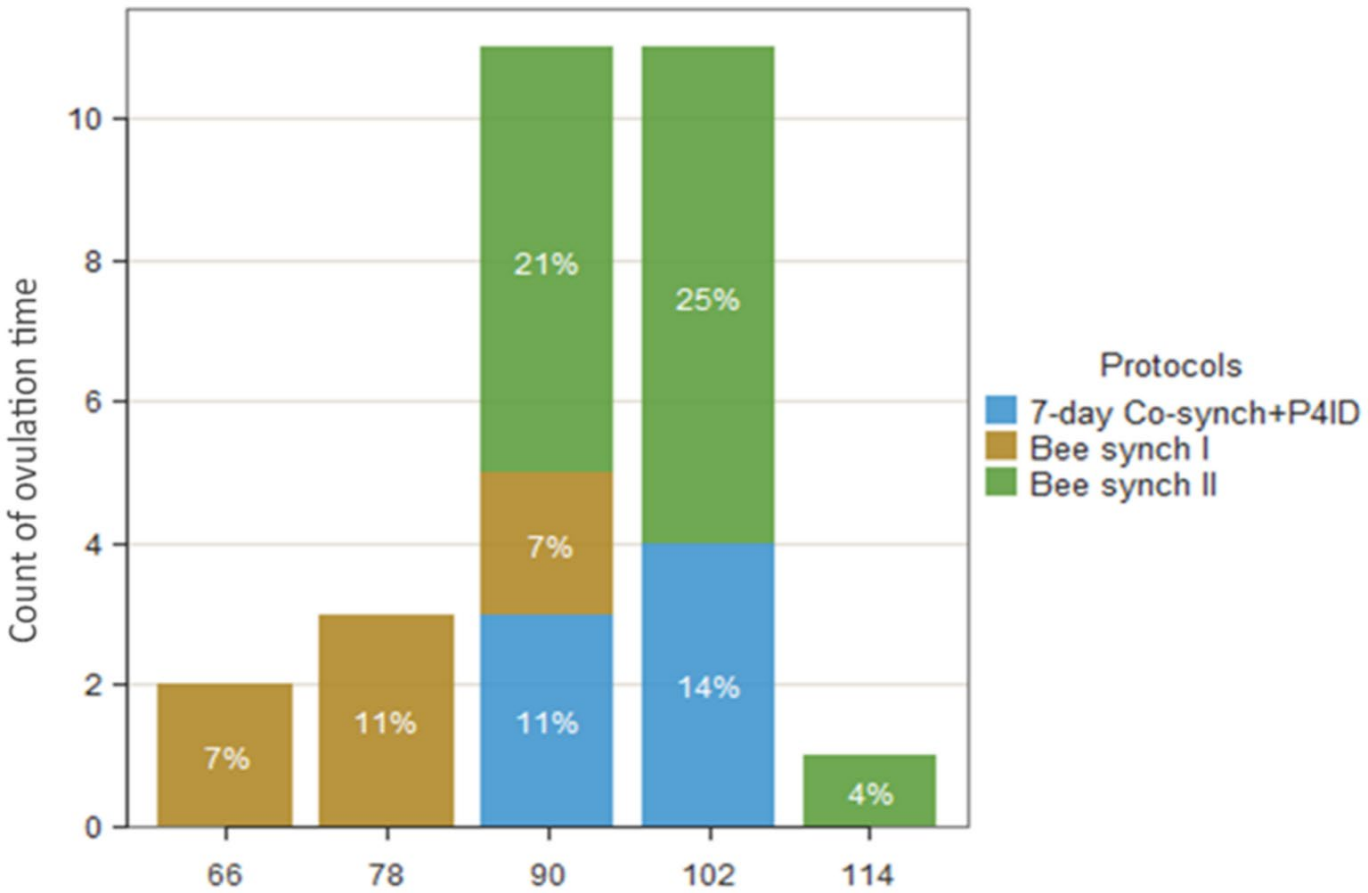
Variation of ovulation time of synchronized cows with reference to time of P4ID withdrawal. Majority of the cows ovulated between 90 and 100 h after P4ID withdrawal. Most of the cows in the Bee synch I group had shorter ovulation times ranging from 66 to 90 h while it ranged from 90 to 114 h in the Bee synch II group. ovulation time was more synchronized and ranged from 90 to 102 h in the 7-day Co-synch + P4ID protocol

**Fig. 6:**
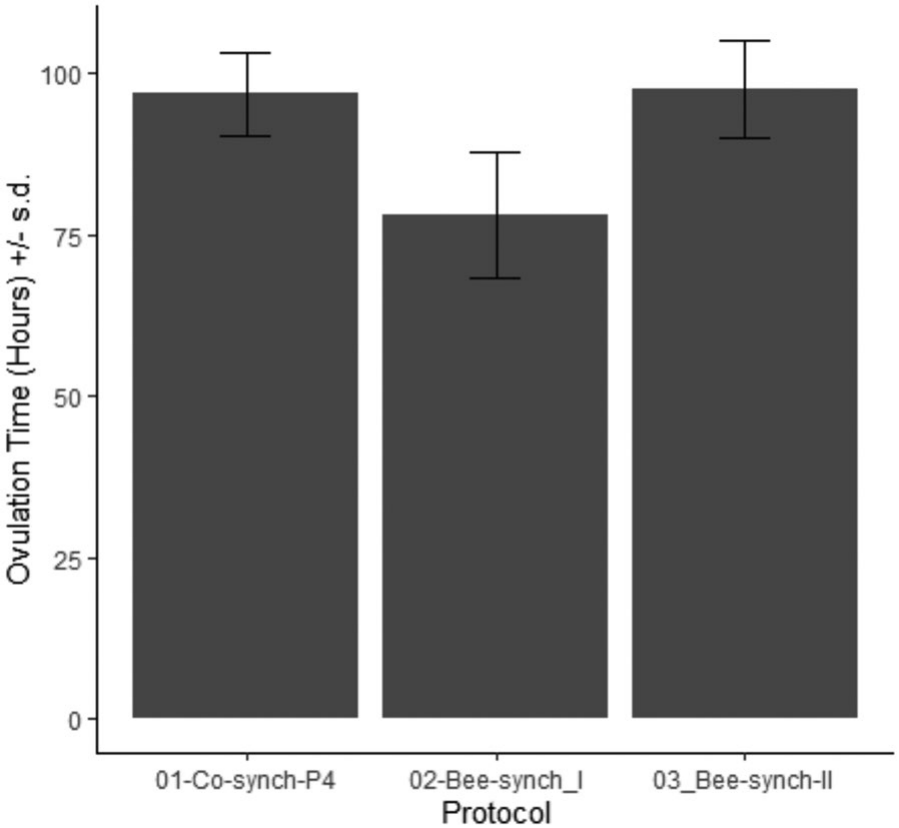
Mean ovulation time across the three estrus and ovulation synchronization protocols. There was a shorter mean ovulation time for Bee synch I protocol as compared to either 7-day Co-synch + P4ID or Bee synch II protocols groups

Generally, majority of the cows ovulated between 90 and 100 h after P4ID withdrawal. Most of the cows in the Bee synch I group had shorter ovulation times ranging from 66 to 90 h while it ranged from 90 to 114 h in the Bee synch II group. Ovulation time was more synchronized and ranged from 90 to 102 h in the 7-day Co-synch + P4ID protocol (Fig. 5). The mean ovulation times for the three synchronization protocols is presented in Table 2 and Fig. 6. While there were no significant differences in ovulation time between 7-day Cosynch + P4ID and Bee synch II groups (P.adj = 0.851), ovulation time was significantly shorter by 19.4% (18.86 h) in the Bee synch I than 7-day Cosynch + P4ID group (ratio = 0.806, SE = 0.071, z = −3.766, P.adj < 0.001). Similarly, ovulation time was significantly longer by 25% (19.7 h) in the Bee synch II group compared to the Bee synch I (time ratio = 1.25, SE = 0.063, z = − 4.451, P.adj < 0.001; Table 5).

### Last GnRH dose to ovulation interval

**Fig. 7:**
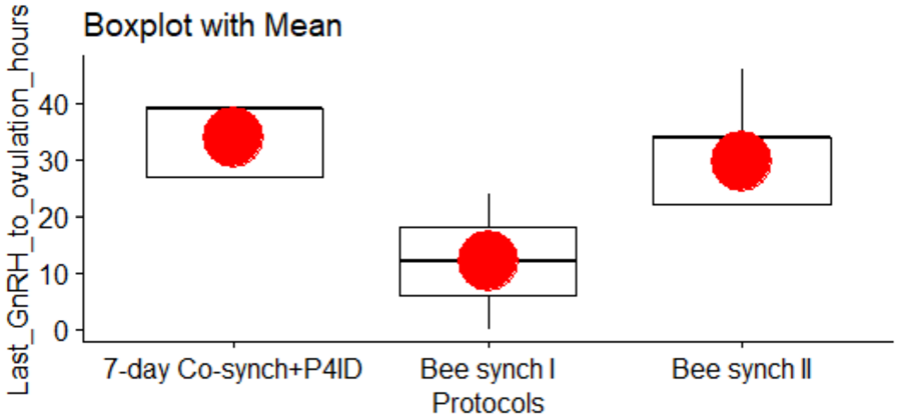
Variation of the ‘last GnRH to ovulation interval’. The mean ‘last GnRH to ovulation interval’ was shorter in the Bee synch I protocol than 7-day Co-synch + P4ID and Bee synch II protocols

The post P4ID withdrawal GnRH dose was administered after 66 h for cows in the 7-day Co-synch group, and 63 h after for the Bee synch I and Bee synch II groups. The mean ‘last GnRH to ovulation interval’ varied across the protocols (Table 2 and Fig. 7). The last GnRH to ovulation interval was by 1.34 times longer for primiparous cows compared to pluriparous cows (time ratio = 1.34, SE = 0.193, z = 2.011, P = 0.044), implying that GnRH induced ovulation time in pluriparous cows is shorter and more favourable. Pairwise comparisons of treatment protocols revealed significant differences in last GnRH dose to ovulation intervals. Compared to Bee synch I, cows under 7-day Cosynch + P4ID exhibited a 3.09-fold increase in the estimated mean Last GnRH to ovulation interval (time ratio = 3.089, SE = 0.645, *z* = 5.407, P.adj < 0.001). Similarly, cows under Bee synch II had a 2.64-fold increase relative to Bee synch I (time ratio = 2.639, SE = 0.492, *z* = 5.206, P.adj < 0.001). However, there was no statistically significant difference between 7-day Cosynch + P4ID and Bee synch II (rate ratio = 0.854, SE = 0.139, *z* = − 0.968, P.adj = 0.333; Table 5).

### Estrus onset to ovulation interval

**Fig. 8:**
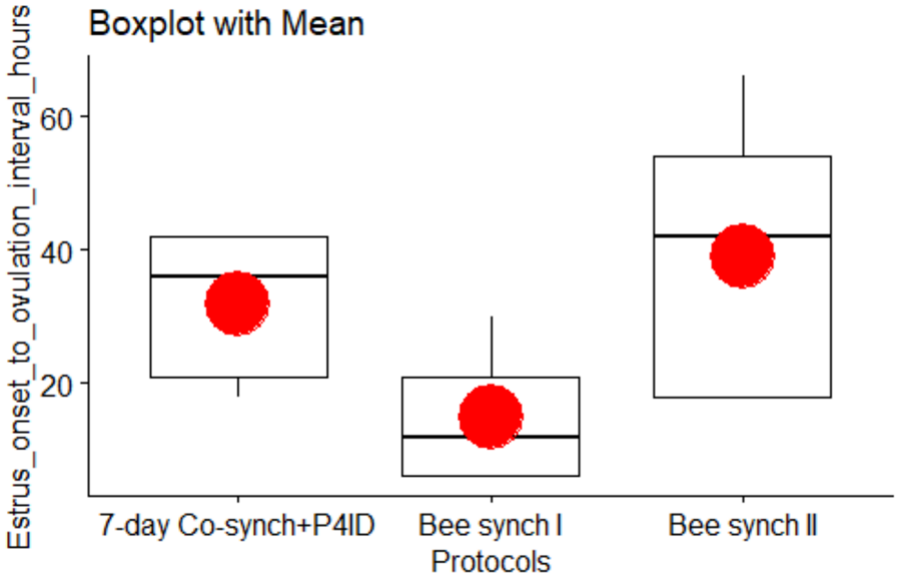
Variation of ‘Estrus onset to ovulation interval’ (hours) for the three synchronization protocols. Estrus onset to ovulation interval is significantly loner for both Bee synch II and 7-day Co-synch + P4ID protocols compared to Bee synch I protocol

The mean estrus onset to ovulation intervals for the protocols are presented in Table 2 and Fig. 8. Cows in 7-day Cosynch + P4ID had by approximately 2 times significantly longer intervals compared to those in Bee synch I (time ratio = 2.13, SE = 0.707, P.adj = 0.0446). Similarly, cows under Bee synch II exhibited significantly longer (2.6 times) intervals than those under Bee synch I (time ratio = 2.60, SE = 0.818, P.adj = 0.0072). However, no significant difference was observed between Bee synch II and 7-day Cosynch + P4ID (time ratio = 1.22, SE = 0.323, P.adj = 0.4547; Table 5). These findings suggest that Bee synch I may lead to a shorter and potentially more favorable estrus-to-ovulation timing.

## Discussion

The finding in our study, that in SHZ, DF diameters generally increased from the time of progesterone withdrawal up to ovulation time is consistent with the findings in cows of *Bos indicus* origin [15], however, when compared at similar times relative to times of progesterone withdrawal, the sizes of dominant follicles in their study were consistently higher than in our study. Differences in DF diameter can be attributed to breed effect and the random stages of the estrous cycle at which synchronization treatments were instituted in our study. The wide variability between *Bos indicus* breeds were further illustrated in our study by the DF diameter at time of last GnRH injection.

Dominant follicles of *B. taurus* cows have been reported to attain ovulatory capacity when they reach 10 mm diameter [34], much larger than the 7 mm obtained in our study and in other studies in *B. indicus* heifers [24]. While we acknowledge that *Bos indicus* generally ovulate smaller follicles, and develop smaller corpora lutea, they have been shown to have greater circulating concentrations of estradiol and progesterone than *B. taurus* [35]. This phenomenon was thought to be related to greater circulating cholesterol, a precursor for estradiol and progesterone, insulin and insulin-like growth factor-1 (IGF-1) in *B. indicus* than in *B. taurus* cows. It can also be argued that fertility of SHZ can be as good as that of *Bos taurus* if the challenge of silent and or weak estrus is addressed. This argument is further affirmed in this study by the finding of a generally higher ovulation rate than estrus expression rate irrespective of protocol studied. Since most SHZ are reared in extensive farming systems where heat detection is almost impractical, Co-sych + P4ID protocols provided a perfect solution for getting silent heat animals to be inseminated and hence opportunity for increased pregnancy rates.

Just like in other studies, we were able to confirm that DF diameters after P4ID withdrawal vary with synchronization protocols [36] and that the performance of the Bee synch I protocol on this aspect was underwhelming compared to the Bee Synch II or 7-day Consynch + P4ID protocols, which is a surprising scenario. The PG dose at P4ID insertion and a double PG at P4ID withdrawal in the Bee synch I protocol would be expected to remove the effect of the CL during these times, keeping the endogenous circulating levels of progesterone at sub-luteal levels and thereby promoting increased rate of follicular growth and ovulatory response [36]. However, the finding that Day 5 CL diameter was highest for the Bee synch II group and least for the Bee synch I group serves to confirm the suggestion that preovulatory DF diameters may have an influence on the resultant CL size [35]. Moreover, the mean DF diameters in our study were higher for both 7-day Co-synch + P4ID and Bee synch II groups for all the times of measurement from the time of P4ID withdrawal.

The finding in our study, that preovulatory DF diameters significantly increased with increase in BCS is in support of the reports by other studies [18, 37], that demonstrated the detrimental effects of low BCS in bovine reproductive performance. Cows in low BCS are in negative energy balance [38, 39], a physiologic status that is associated with decreased insulin and IGF-1, and consequently an increased plasma growth hormone concentration which reduces the levels of leptin. Leptin is crucial in the secretion of estrogen and progesterone, and promoting follicular growth, development and ovulation [40].

The DF growth and therefore preovulatory DF sizes were expected to be higher in Bee Synch I group of cows because all luteal tissue had been eliminated by the first PG as well as the double injection of PG at P4ID withdrawal. The exogenous GnRH in 7-day Co-synch + P4ID and Bee Synch I protocols altered follicular development by inducing luteinization and (or) ovulation of dominant follicles. The double PG injection in Bee Synch I is expected to guarantee complete lysis of all luteal tissue and absolute removal of the progesterone block on the hypothalamus and pituitary. This, together with second GnRH acted on the pituitary to induce release of FSH and LH hence permitting uninhibited yet synchronized growth of new follicles at a faster rate than the other two protocols that did not have a complementary PG [14]. Since Bee Synch II did not have an initial luteotropic and ovulatory GnRH and only one PG injection, the growth of follicles subsequent to P4ID withdrawal would have been slowed by remnant luteal tissue. However, Bee synch II protocol performed better than Bee synch I in regards to DF diameters at all times of measurements, from P4ID withdrawal to preovulation. The elimination of the first GnRH dose in the Bee synch II protocol seems to have averted the chances of development of immature CLs which would be around day 5, and thereby minimized the circulating levels of progesterone which would have inhibited DF growth by negative feedback on the hypothalamus and anterior pituitary gland. Bee synch II protocol is able to achieve this while concurrently eliminating the requirement for double dose of PG and generally reducing the costs involved in the GnRH [14, 17].

Whereas the mean estrus expression rate across the three protocols in the SHZ cows was low and comparable to studies in other *Bos indicus* breeds elsewhere [41], the finding that Bee synch II registered the highest and Bee synch I protocol the lowest estrus expression rate gives Bee synch II additional comparative advantage over the other two protocols. It is interesting to observe that the onset of estrus on average was similar across the three protocols tested, and coincided with the time of the second injection of GnRH in all the three protocols, affirming their general appropriateness in optimizing conception in the SHZ.

The observation in this study that ovulation rate was higher than the estrus expression rate irrespective of the protocol is not surprising, given that these protocols aim at inducing ovulation and not necessarily causing heat expression, and that is why fixed time AI is a requirement. This observation is also a confirmation that SHZ in Uganda just like other *Bos indicus* cows naturally experience silent estrus, and low estrus signs [8]. This finding also further emphasizes the importances of fixed-time synchronization protocols for the *B. indicus* cattle that naturally experience high rates of silent estrus.

We have demonstrated a significant variation in ovulation rate based on type of estrus and ovulation synchronization protocol in the SHZ of Uganda, with the 7-day Co-synch + P4ID protocol registering the lowest ovulation rate and the Bee synch II protocol the highest. The poor ovulatory response recorded in the current study for cows in the 7-day Co-synch + P4ID and Bee synch I groups could be as a result of small DF sizes at the time of last GnRH dose. Small follicles have been reported to lack messenger RNA (mRNA) and their LH receptor proteins are nonresponsive to GnRH [42]. Indeed, the DF diameters at time of last GnRH injection in our study were significantly higher in the Bee synch II group than in the Bee synch I group. Since it has been argued that ovulation induction by use of GnRH is typically effective only when administered after occurrence of follicular deviation and establishment of dominance [43], further studies are necessary to demonstrate the effect of delaying the time of last GnRH administration on the ovulatory capacity of DF. Regarding the 11.7% of the cows that expressed estrus but did not ovulate, a similar trend has been documented in Nellore cows synchronized with Ovsynch and prostaglandin protocols where estrus rates of 87% and 78% respectively, yielded only a 50% ovulation rate [44]. The failure to ovulate could be explained by the immature size of the dominant follicles that did not express LH receptor proteins at the time of GnRH dose and thus did not respond by ovulation.

The mean ovulation time from P4ID withdrawal and at the time of last GnRH injection was lowest for the Bee synch I protocol, similar to that reported in Nellore cows treated with 7-day progesterone implant protocols involving estradiol treatments at 3 different times relative to progesterone withdrawal [22]. On the other hand, the 7-day Co-synch + P4ID and Bee synch II groups recorded ‘last GnRH to ovulation’ intervals similar to that reported for Norwegian Red cows synchronized with two doses of PGF_2_α 11 days apart followed with a dose GnRH two days later [45], and for Sahiwal cows in another study [44].

The interval of 64–68 h and 60–66 h recommended for timed AI after P4ID removal in the Bee synch II and 7-day Co-synch protocols respectively is much earlier than the ovulation times recorded in these two protocols. This presents an opportunity for further modification of these protocols to enhance their efficiency and conception rates in SHZ cows in Uganda. Whereas the Bee synch I protocol performed least among the three in the SHZ cows, it also presents an opportunity of studying this protocol further, with the view to adjusting the timing of hormone treatments to take advantage of critical stages in the estrous cycle, like timing and threshold DF sizes that are responsive to LH surge. Although estrus expression and ovulation were poor in the Bee synch I protocol group, AI is prescribed to take place concurrent with GnRH injection at 64–68 h from time of P4ID withdrawal, and we found a mean ovulation time of 78.0 ± 2.6 h for this protocol in the SHZ cows. Given that the best time for conducting AI is 7–18 h before ovulation [45], the Bee synch I protocol had the best performance in this regard compared to Bee synch I and 7-day Co-synch + P4ID. Bee synch I protocol could therefore have a better conception rate in SHZ cows if applied as prescribed.

## Conclusions

The Bee synch II protocol performed the best in most of the aspects studied, followed by the 7-day consych + P4ID and the Bee synch I, performed the least. In all the three protocols, further studies are needed to enhance their efficiency and conception rates in SHZ cows. The significant differences in preovulatory follicle diameter between protocols in the current study suggest a difference in effectiveness of estrous synchronization protocols, with the Bee synch II and the 7-day Co-synch + P4ID being more effective than Bee synch I protocol. Larger preovulatory DF diameters were recorded with 7-day Co-synch + P4ID and Bee synch II protocols than with Bee synch I protocol and a generally low estrous expression rate was recorded, underscoring the importance of fixed-time estrous synchronization protocols over protocols that rely on estrus detection in the SHZ cattle which are naturally characterized by poor estrus expression. Protocol modifications are recommended with regards to times of last GnRH administration and insemination taking into consideration the optimum timing for insemination relative to the time of ovulation to achieve better effectiveness in SHZ cattle. Studies to ascertain conception rates of SHZ cows due to 7-day Co-synch + P4ID, Bee synch I and Bee synch II protocols are recommended. We can confirm that there are indeed differences in ovarian follicular response to three Co-Synch + P4ID fixed-time estrus and ovulation synchronization protocols, which point to variation in their effectiveness in the reproductive management of *Bos indicus*, SHZ cattle in Uganda.

## Data Availability

The datasets used and/or analyzed during the current study are available from the corresponding author on reasonable request.

## Abbreviations

AI: Artificial insemination
AIC: Akaike information criterion
BCS: Body condition score
BIC: Bayesian information criterion
CL: Corpus luteum
DF: Dominant follicle
GnRH: Gonadotropin-releasing hormone
IGF-1: Insulin-like growth factor -1
OR: Odds ratio
P4ID: Progesterone-releasing intravaginal device
PGF_2_α: Prostaglandin F_2_α
PRID: Progesterone-releasing intravaginal device
SHZ: East African shorthorn zebu
